# Early diagnosis and meta-agnostic model visualization of tuberculosis based on radiography images

**DOI:** 10.1038/s41598-023-49195-x

**Published:** 2023-12-20

**Authors:** Sasikaladevi Natarajan, Pradeepa Sampath, Revathi Arunachalam, Vimal Shanmuganathan, Gaurav Dhiman, Prasun Chakrabarti, Tulika Chakrabarti, Martin Margala

**Affiliations:** 1 Department of Computer Science and Engineering, School of Computing, SASTRA Deemed University, Thanjavur, Tamil Nadu 613401 India; 2 Department of Information Technology, School of Computing, SASTRA Deemed University, Thanjavur, Tamil Nadu 613401 India; 3 Department of Electronics and Communications Engineering, School of EEE, SASTRA Deemed University, Thanjavur, Tamil Nadu 613401 India; 4 Deep Learning Lab, Department of Artificial Intelligence and Data Science, Ramco Institute of Technology, Rajapalayam, Tamil Nadu India; 5 School of Sciences and Emerging Technologies, Jagat Guru Nanak Dev Punjab State Open University, Patiala, 147001 India; 6 Department of Electrical and Computer Engineering, Lebanese American University, Byblos, Lebanon; 7 Department of Computer Science and Engineering, University Centre for Research and Development, Chandigarh University, Gharuan, Mohali, 140413 India; 8 Department of Computer Science and Engineering, Graphic Era Deemed to be University, Dehradun, 248002 India; 9 Division of Research and Development, Lovely Professional University, Phagwara, 144411 India; 10 Centre of Research Impact and Outreach, Chitkara University Institute of Engineering and Technology, Chitkara University, Punjab, India; 11 Sir Padampat Singhania University, Udaipur, Rajasthan India; 12 University of Louisiana at Lafayette, Lafayette, USA

**Keywords:** Diseases, Medical research

## Abstract

Despite being treatable and preventable, tuberculosis (TB) affected one-fourth of the world population in 2019, and it took the lives of 1.4 million people in 2019. It affected 1.2 million children around the world in the same year. As it is an infectious bacterial disease, the early diagnosis of TB prevents further transmission and increases the survival rate of the affected person. One of the standard diagnosis methods is the sputum culture test. Diagnosing and rapid sputum test results usually take one to eight weeks in 24 h. Using posterior-anterior chest radiographs (CXR) facilitates a rapid and more cost-effective early diagnosis of tuberculosis. Due to intraclass variations and interclass similarities in the images, TB prognosis from CXR is difficult. We proposed an early TB diagnosis system (tbXpert) based on deep learning methods. Deep Fused Linear Triangulation (FLT) is considered for CXR images to reconcile intraclass variation and interclass similarities. To improve the robustness of the prognosis approach, deep information must be obtained from the minimal radiation and uneven quality CXR images. The advanced FLT method accurately visualizes the infected region in the CXR without segmentation. Deep fused images are trained by the Deep learning network (DLN) with residual connections. The largest standard database, comprised of 3500 TB CXR images and 3500 normal CXR images, is utilized for training and validating the recommended model. Specificity, sensitivity, Accuracy, and AUC are estimated to determine the performance of the proposed systems. The proposed system demonstrates a maximum testing accuracy of 99.2%, a sensitivity of 98.9%, a specificity of 99.6%, a precision of 99.6%, and an AUC of 99.4%, all of which are pretty high when compared to current state-of-the-art deep learning approaches for the prognosis of tuberculosis. To lessen the radiologist’s time, effort, and reliance on the level of competence of the specialist, the suggested system named tbXpert can be deployed as a computer-aided diagnosis technique for tuberculosis.

## Introduction

**Figure 1:**
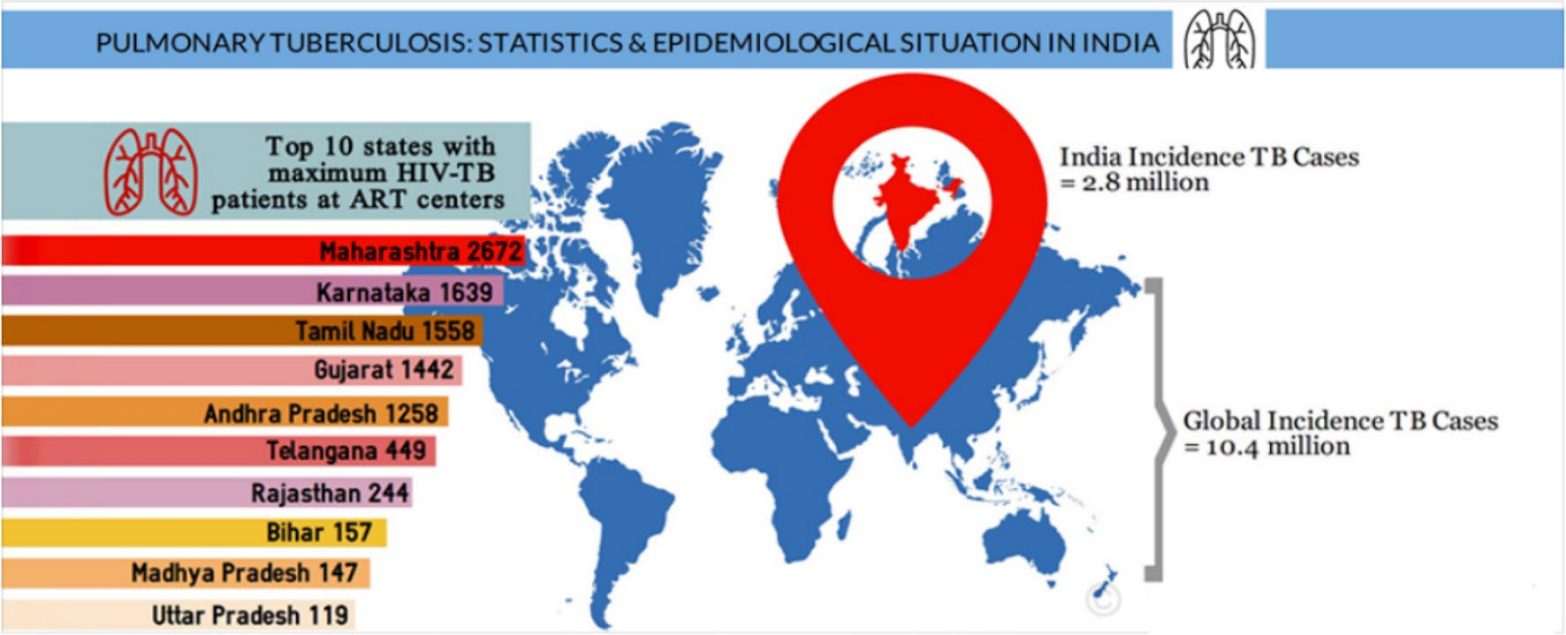
Indian states’ TB statistics reported by Medindia^3^.

The emitted airborne microdroplets enable the tuberculosis-causing bacteria to spread from one person to another. Patient with TB infection is usually asymptomatic and requires a long medical course for complete treatment. Tuberculosis is the most prevalent infectious disease in the world despite being wholly preventable and treatable^1^. In the National Tuberculosis Elimination Program, the government gathers most of the TB statistics for India (previously called the RNTCP), and the World Health Organization (WHO) receives this data. According to WHO TB statistics, there will likely be 2,590,000 million cases of TB in India in 2021, and it afflicts 6 percent of children aged 0 to 14 years, 36 percent of women, and 58% of men. This translates to a rate of 188 per 100,000 people^2^. Figure 1 portrays the Indian states' TB statistics reported by Medindia.

Early diagnosis of TB reduces the mortality rate and prevents infection from spreading to others^4^. Frequent screening is required for high-risk groups to prevent infection^5^.

The sputum culture test, which takes a long time and is frequently unreliable, is one of the conventional TB diagnosis methods^6^. Rapid TB tests can produce results in as little as 24 h, while other lung disorders might take up to 8 weeks to produce results. The main challenging task of a test is to produce a sample size large enough for it to be tested. For this test, saliva from the upper airways is useless. Posterior-anterior (PA) view of chest radiographs (CXR) is an affordable and fast method for screening TB^7^. However, using CXR for TB diagnosis is tedious due to interclass similarity and intraclass variation in the low-radiation CXR images.

Moreover, the availability of radiologists for CXR annotations is also less in developing countries like India. Hence, there is a demand for including artificial intelligence (AI) for TB’s accurate and fast prognosis in CXR images^8^. The WHO has suggested CXR screening as a method of TB case detection. It can determine the location, type, and severity of lesions and check for any lesions that might be TB-related right away. Previous research revealed that case finding based on CXR testing was more accurate than case finding based on symptoms.

In addition to using computer-aided detection (CAD) systems to read medical images, several CAD software solutions have been developed to analyze digital CXRs for anomalies suggestive of TB. Deep learning methods are playing a vital role in medical image-based diagnostic systems. Deep learning approaches are constructed on Convolutional Neural Networks (CNN), which comprise deep feature extractors. When the CAD system is developed to diagnose TB, that system should be trained by a large dataset, including noisy images. The primary goal of the diagnostic system is to interpret chest X-ray images with high accuracy compared to radiologists. There needs to be more radiologists in a densely populated country like India. Therefore, developing an AI-based tool for TB prognosis from chest X-ray images that accurately replicate the radiologist is necessary.

The contributions of this article are highlighted below:

- The linear interpolation method is proposed to decrease intra-class variation and inter-class similarity for minimal misclassification error.
- Enhances the quality of the low-radiation chest X-ray images by fusing the original image with the interpolation overlay map.
- Fine-tuned the layers of deep inception residual neural network, and then Adam optimizer is used to train the network.
- Attained the highest accuracy, sensitivity, and specificity for the benchmark datasets.

The rest of the paper is organized as follows. The related works are discussed in Sect. “Related work”. The proposed framework for accurately diagnosing tuberculosis from radiographic pictures based on fused linear triangular interpolation is shown in Sect. “Proposed framework”, and the experiments, data collecting, data analysis, and experimental findings and discussion are shown in Sect. “Results”. Section “Conclusion” concludes and discusses the future direction. In the domain of deep learning-based image classification, many methods are proposed by researchers in the form of transfer learning, reinforcement learning, and meta-learning. A significant challenge in this domain is reducing the misclassification error. Only a few researchers addressed this. In this paper, we have proposed the linear interpolation-based technique to resolve the misclassification error by reducing the inter-class similarities and intra-class variation. This is a novel approach in the classification domain.

## Related work

Medical image analysis is essential in diagnosis, severity analysis, and treatment processes in the medical field. For decades, the researcher has used chest X-ray images to diagnose tuberculosis (TB). Deep learning-based image analysis is the paradigm shift in the computer vision and pattern recognition problem. Since the deep learning algorithm^9–30^ is more precise, it can effectively train medical images.

Recently, there have been many more commercially accessible CAD systems with Deep Learning technology. They have been demonstrated to be extremely sensitive and potentially prevent the need for many of the ensuing confirmation procedures. However, TB's prevalence may significantly impact Deep Learning (DL) system performance at similar thresholds. This section analyses the deep learning-based diagnosis methods of TB alone for better comparison with other component algorithms in this domain. To diagnose TB using chest X-ray images, the researchers proposed several deep-learning and machine-learning techniquestabulated in Table 1.

**Table 1:** Existing methods of AI-based solutions for the interpretation of digital CXRs.

Ref	Classification	Dataset	Accuracy
^9^	Support vector machine, deep convolutional neural network,	Dr. Peinado –Peruvian: 453-Normal, 4248- TB	89
^10^	Random forest and extremely randomized trees	Private:73 –TB, 319-Normal	84
^11^	Support vector machine + multilayer perceptron neural networks (SVNN), back propagation neural networks	Private:1278-TB, 259-Normal	92.5
^12^	Transfer learning: AlexNet	Dr. Peinado –Peruvian: 453-Normal, 4248- TB	85.68
^9^	Deep convolutional neural network	Dr. Peinado –Peruvian: 453-Normal, 4248- TB	89.6
^13^	Ensemble:AlexNet and GoogleNet	HIPAA-compliant datasets: 707-Normal, 300-TB	95.3
^14^	Pre-trained Network: ResNet – Feature extraction Classification—Support Vector Machine	Montgomery dataset: 80-Normal, 58-TB, Shenzhen dataset: 326-Normal, 336-TB	83.4
^15^	Artificial neural networks with Adam optimizer	Montgomery dataset: 80-Normal, 58-TB, Shenzhen dataset: 326-Normal, 336-TB	82.09
^16^	SegNet	Private: 73 –TB, 319-Normal	84.3
^17^	Pre-trained network: GoogleNet	Montgomery dataset: 80-Normal, 58-TB, Shenzhen dataset: 326-Normal, 336-TB	90
^18^	Pre-trained neural network: VGG16, DenseNet and ResNet	Montgomery dataset: 80-Normal, 58-TB, Shenzhen dataset: 326-Normal, 336-TB	85
^19^	Pre-trained neural network: VGG16, Inception v4, ResNet and Resnet-Inception v2	Montgomery dataset: 80-Normal, 58-TB, Shenzhen dataset: 326-Normal, 336-TB	96.1
^20^	Ensemble: ResNet, Inception-ResNet and DenseNet	Montgomery dataset: 80-Normal, 58-TB, Shenzhen dataset: 326-Normal, 336-TB	90.5
^21^	Ensemble: AlexNet, GoogleNet and ResNet	Montgomery dataset: 80-Normal, 58-TB, Shenzhen dataset: 326-Normal, 336-TB	88.24
^22^	Probabilistic neural network	Private:105-Normal, 105-TB	96
^23^	Pre-trained deep learning CNN: ResNet18, ResNet50, ResNet101, DenseNet201, InceptionV3, VGG19, ChexNet, SqueezeNet, and ResNet50	Datasets from the NLM, Belarus, NIAID, and RSNA: 3500—Normal, 3500 – TB	98.6
^24^	Feature extraction: Gabor filter &inception v3 Classification: logistic regression	Montgomery dataset: 80-Normal, 58-TB, Shenzhen dataset: 326-Normal, 336-TB	83.23
^25^	Segmentation, support vector machine classifier	Montgomery dataset: 80-Normal, 58-TB, Shenzhen dataset: 326-Normal, 336-TB	98
^26^	Feature extraction: MobileNet Classification: Convolutional neural network	Montgomery dataset: 80-Normal, 58-TB, Shenzhen dataset: 326-Normal, 336-TB	94.1
^27^	Ensemble- deep convolutional neural network	Montgomery dataset: 80-Normal, 58-TB, Shenzhen dataset: 326-Normal, 336-TB	92.8
^28^	SegmentationEnsemble transfer learning-VGG, inception v3	MIMIC: 370-Normal, 0-TB, Montgomery dataset: 80-Normal, 58-TB, Shenzhen dataset: 326-Normal, 336-TB, Generated: 1000-TB, 0-Normal	97.1
^29^	High-frequency emphasis filtering, Unsharp masking (UM), Pre-Trained ResNet, and EfficientNet (HEF)	Montgomery dataset: 80-Normal, 58-TB, Shenzhen dataset: 326-Normal, 336-TB	93.81
^30^	Deep convolutional neural network	Montgomery dataset: 80-Normal, 58-TB, Shenzhen dataset: 326-Normal, 336-TB	91

As shown in Table 1, different deep-learning methods have been applied to various datasets to diagnose tuberculosis. Only a few techniques, such as feature extraction and classification, are based on a two-phase paradigm. In Ref.^11,24^, signal processing-based feature extraction methods are applied, including the Gabor filter, Speeded Up Robust Features(SURF), and Local binary patterns (LBP). Pre-trained deep learning networks extract deep features in Ref.^14,26^. Support Vector Machine (SVM) and Bayesian optimization-based classifiers are used in Ref.^9,11,25,28,31,32^.

Deep convolutional neural networks (DCNN) trained by the Imagenet dataset are used to retrain the network with the TB dataset. For TB diagnosis, pre-trained neural networks such as GoogleNet, VGG16, ResNet, Inception V3, Resnet-Inception V2, DenseNet, SqueezeNet, and MobileNet^17–19,23,33^. In machine learning algorithms, transfer learning allows the use of feature representations from a model that has already been trained rather than having to create a new model from the beginning. For the classification of TB, the transfer learning-enhanced pre-trained AlexNet model is considered^12^.

For TB diagnosis, a variety of ensemble techniques are utilized. The images extract features using Alexnet and Google^12^. ResNet, Inception-Resnet, and DenseNet are used for classification^20^. An ensemble of ResNet, GoogleNet, and AlexNet is used in Ref.^21^. An ensemble of VGG and Inception V3 is used in Ref.^28^. The most challenging task of X-ray-based diagnosis is the quality of the image. As it is based on low and varying radiation, there is a variation in the quality of the image.

Moreover, it is also challenging to handle interclass similarity and intraclass variation in the images. There is a demand to customize the X-ray images to provide better prediction performance. Segmenting the infected region and applying a deep learning model will perform better than normal images. Nevertheless, segmentation requires ground truth images unavailable for benchmark TB datasets. Another drawback of segmentation is the additional overhead. Enhancing the Region of Interest (ROI) will improve feature extraction from the TB chest X-ray images.

## Proposed framework

**Figure 2:**
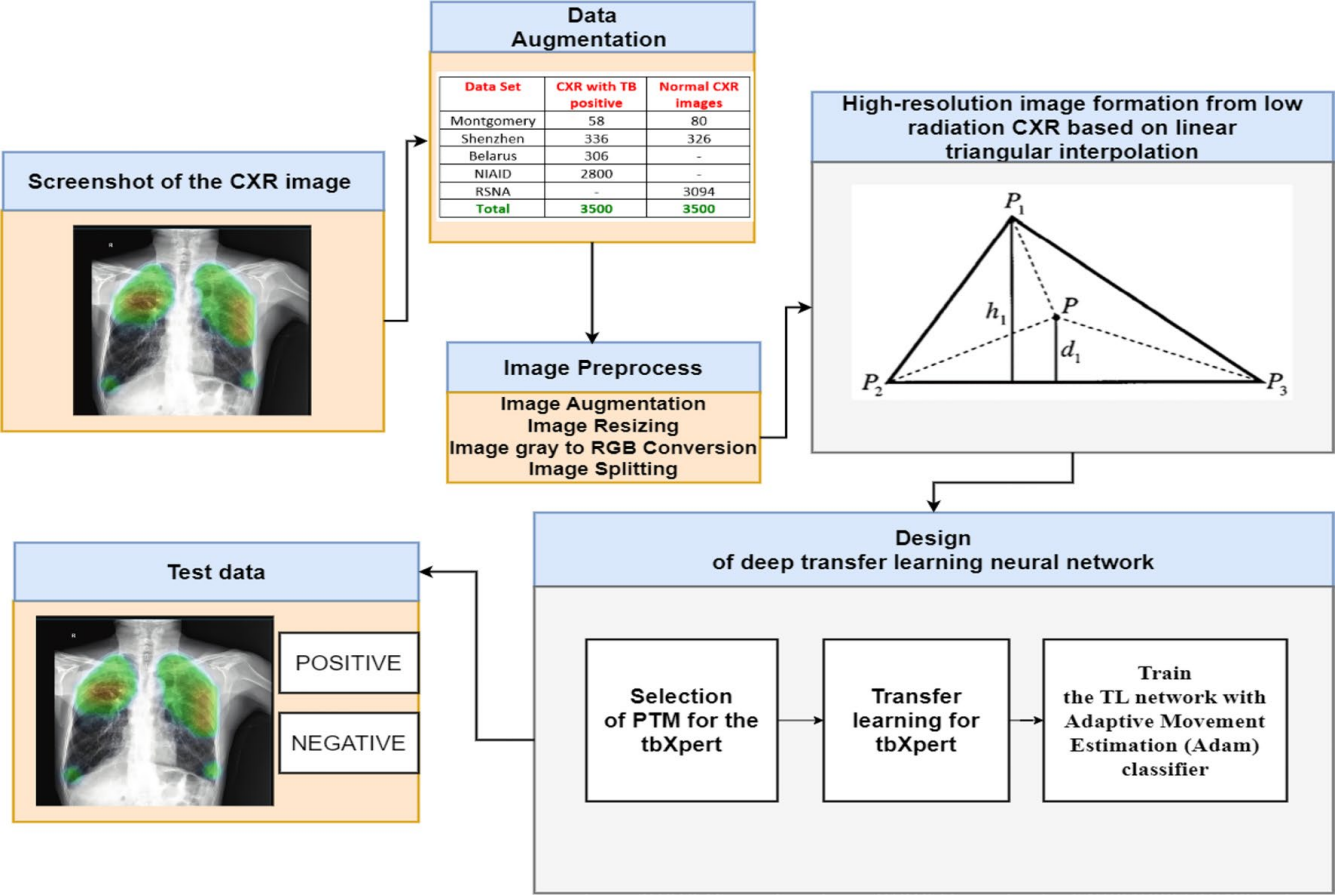
tbXpert architecture.

The proposed diagnosis system named tbXpert is designed following the three significant steps: data augmentation, creating deeply fused X-ray images using Linear Triangular Interpolation (FLT), and deep transfer learning. All this processing was realized in MATLAB 2020b with image processing and deep learning toolboxes. Figure 2 portrays the tbXpert system with different steps.

### Data augmentation

The four benchmark available datasets for TB—include the National Library of Medicine (NLM)^34^, Belarus^35^, the National Institute of Allergy and Infectious Disease (NIAID)^35^, and the Radiological Society of North America (RSNA)^36^—are used to train and test the suggested diagnosis system. The total number of images from four benchmark datasets is displayed in Table 2.

**Table 2:** CXR dataset summary.

Data set	CXR with TB-positive	Normal CXR images
Montgomery	58	80
Shenzhen	336	326
Belarus	306	–
NIAID	2800	–
RSNA	–	3094
Total	3500	3500

#### Belarus

306 chest X-ray images of 169 patients with a resolution of 2248 × 2248 pixels were published in a dataset by the Ministry of Health, Republic of Belarus. It has only TB-infected X-ray images.

#### NLM

It published two chest X-ray datasets for TB, such as Montgomery and Shenzhen. The 58 chest X-ray images in the Montgomery dataset have the label "TB" and the remaining 80 have the label "normal”. The resolution of the images is 4020 × 4892.

#### Shenzhen

It contains 662 posterior-anterior chest X-ray images, where 336 images with the label TB and the remaining 326 images are labeled normal. The resolution of the images is 3000 × 3000.

#### RSNA

It published the Chest X-ray images for the detection of pneumonia. The 3094 typical chest X-ray images are used from that database.

#### NIAID

It released a dataset of 2800 chest X-ray pictures showing TB positivity. These data were collected from seven different countries around the world.

The original X-ray images can be downloaded in both grayscale and Dicom formats. The gathered images from different sources are preprocessed, and the images are downsized to 299 × 299 pixels.

### Formation of High-resolution from low radiation CXR using linear triangular interpolation method

Since tbXpert is designed for use globally, it is trained and validated using a variety of datasets. The variation in quality, intensity, contrast, and radiological characteristics is the fundamental problem with using different datasets. Different radiological settings cause intraclass variations and interclass similarity. Reduced intraclass variation improves the performance of the tbXpert system. Here, scattered interpolation points are created based on the Region of Interest using the LTI method^37,38^. The original CXR images are overlaid with the scattered interpolated points to enhance the image.

**Figure 3:**
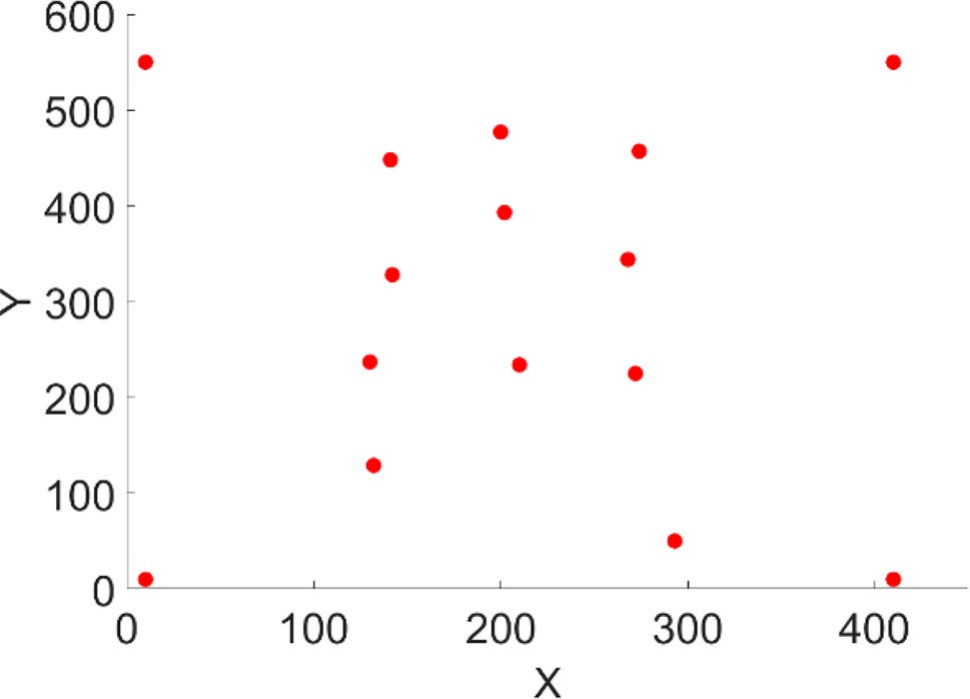
Reference points for CXR.

The region of interest for the TB infection in the chest is displayed on the CXR image based on the reference point in Fig. 3. A piecewise triangular surface is formed from the triangulation of this 2D (x, y) plane, with the points (x,i) (y,i) and (z,i). A triangle-shaped asymmetric network is created when edges connect these triangle-shaped pieces.

**Figure 4:**
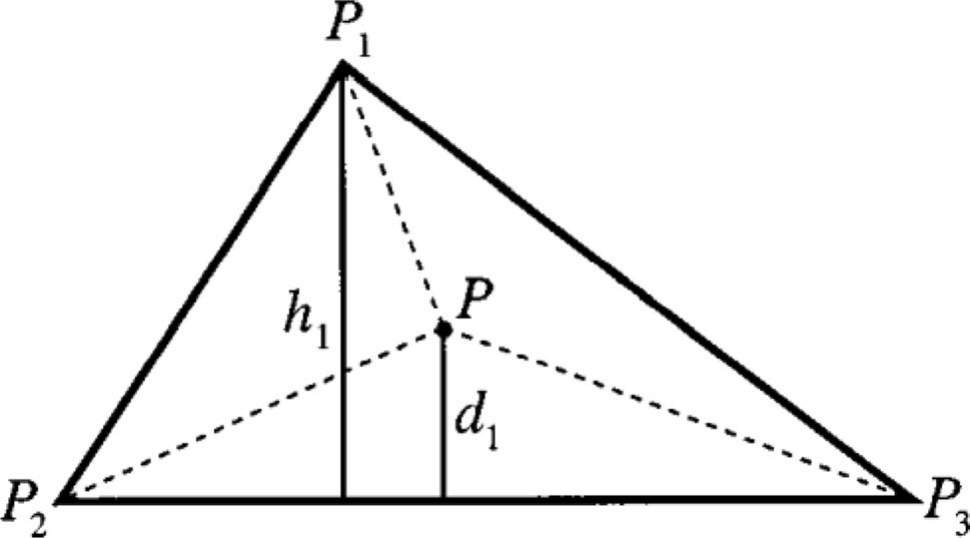
Interpolated point P from P1, P2, and P3.

${P}_{1}=\left({x}_{1},{y}_{1},{z}_{1}\right), {P}_{2}=\left({x}_{2},{y}_{2},{z}_{2}\right)$ and ${P}_{3}=\left({x}_{3},{y}_{3},{z}_{3}\right)$ are the three triangular points.Each triangle is interpolated using the bivariate linear interpolation (BLI) method, as shown in Fig. 4.

Obtain linear equations such as

1 $${z}_{1}=a{x}_{1}+b{y}_{1}+c,$$

2 $${z}_{2}=a{x}_{2}+b{y}_{2}+c,$$

3 $${z}_{3}=a{x}_{3}+b{y}_{3}+c,$$

Undefined co-efficient $(a,b,c)$ are calculated by solving this linear formula. Then, we can calculate any subjective point with the coordinate as $\left(x and\right)$ inside the triangle.

The points from the (x, y) plane must be projected onto the (u, v) plane to interpolate image pixels. Consider the triangle's interpolated point P using the three points P1, P2, and P3 in the affine coordinate system (x, y). ${X}_{1}=({x}_{1}, {y}_{1})$, ${X}_{2}=({x}_{2}, {y}_{2})$, and ${X}_{3}=({x}_{3}, {y}_{3})$ are the affine coordinates of these points.

4 $$X={X}_{1}+\left({X}_{2}-{X}_{1}\right){a}_{2}+\left({X}_{3}-{X}_{1}\right){a}_{3}.$$

The affine system's representation of the interpolated point coordinates is as follows:

5 $$x={x}_{1}+\left({x}_{2}-{x}_{1}\right){a}_{2}+\left({x}_{3}-{x}_{1}\right){a}_{3}\,\,\mathrm{ and }\,\,y={y}_{1}+\left({y}_{2}-{y}_{1}\right){a}_{2}+\left({y}_{3}-{y}_{1}\right){a}_{3}.$$

Henceforth, the matrix form,

6 $$\left(\genfrac{}{}{0pt}{}{x}{y}\right)=\left(\genfrac{}{}{0pt}{}{{x}_{1}}{{y}_{1}}\right)+\left(\begin{array}{cc}{x}_{2}-{x}_{1}& {x}_{3}-{x}_{1}\\ {y}_{2}-{y}_{1}& {y}_{3}-{y}_{1}\end{array}\right)\left(\genfrac{}{}{0pt}{}{{a}_{2}}{{a}_{3}}\right),$$

7 $$\left(\genfrac{}{}{0pt}{}{{a}_{2}}{{a}_{3}}\right)={\left(\begin{array}{cc}{x}_{2}-{x}_{1}& {x}_{3}-{x}_{1}\\ {y}_{2}-{y}_{1}& {y}_{3}-{y}_{1}\end{array}\right)}^{-1}\left(\genfrac{}{}{0pt}{}{x-{x}_{1}}{y-{y}_{1}}\right).$$

Consider the three-point (u, v) coordinates mapping. ${P}_{1}, {P}_{2}$, and ${P}_{3}$. The coordinates of these points are represented as ${U}_{1}=({x}_{1}, {y}_{1})$, ${U}_{2}=({x}_{2}, {y}_{2})$, and ${U}_{3}=({x}_{3}, {y}_{3})$. The point $X=(x,y)$ is mapped onto the point $U=\left(u,\right)$ by using linear DT interpolation (u,v)

8 $$U={U}_{1}+\left({U}_{2}-{U}_{1}\right){a}_{2}+\left({U}_{3}-{U}_{1}\right){a}_{3}.$$

According to the affine system, the interpolated point coordinates are shown as,

9 $$u={u}_{1}+\left({u}_{2}-{u}_{1}\right){a}_{2}+\left({u}_{3}-{u}_{1}\right){a}_{3 } \,\,{\text{and}} \,\,v={v}_{1}+\left({v}_{2}-{v}_{1}\right){a}_{2}+\left({v}_{3}-{v}_{1}\right){a}_{3}.$$

Hence, the matrix form,

10 $$\left(\genfrac{}{}{0pt}{}{u}{v}\right)=\left(\genfrac{}{}{0pt}{}{{u}_{1}}{{v}_{1}}\right)+\left(\begin{array}{cc}{u}_{2}-{u}_{1}& {u}_{3}-{u}_{1}\\ {v}_{2}-{v}_{1}& {v}_{3}-{v}_{1}\end{array}\right)\left(\genfrac{}{}{0pt}{}{{a}_{2}}{{a}_{3}}\right).$$

In the (u,v), the new interpolated picture pixel is calculated by deducting (a2, a3) from (10).

11 $$\left(\genfrac{}{}{0pt}{}{u}{v}\right)=\left(\genfrac{}{}{0pt}{}{{u}_{1}}{{v}_{1}}\right)+\left(\begin{array}{cc}{u}_{2}-{u}_{1}& {u}_{3}-{u}_{1}\\ {v}_{2}-{v}_{1}& {v}_{3}-{v}_{1}\end{array}\right){\left(\begin{array}{cc}{x}_{2}-{x}_{1}& {x}_{3}-{x}_{1}\\ {y}_{2}-{y}_{1}& {y}_{3}-{y}_{1}\end{array}\right)}^{-1}\left(\genfrac{}{}{0pt}{}{x-{x}_{1}}{y-{y}_{1}}\right).$$

**Figure 5:**
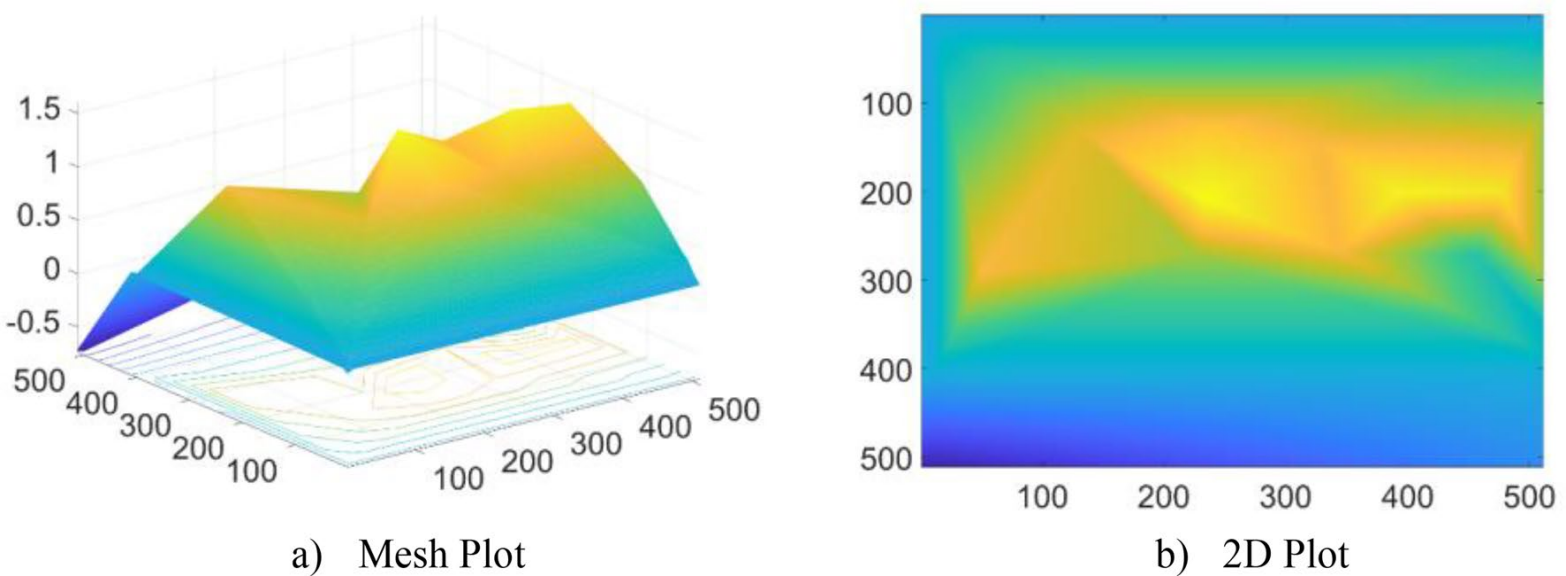
Overlay image based on DT Interpolated points (**a**) Mesh plot and (**b**) 2D plot.

**Figure 6:**
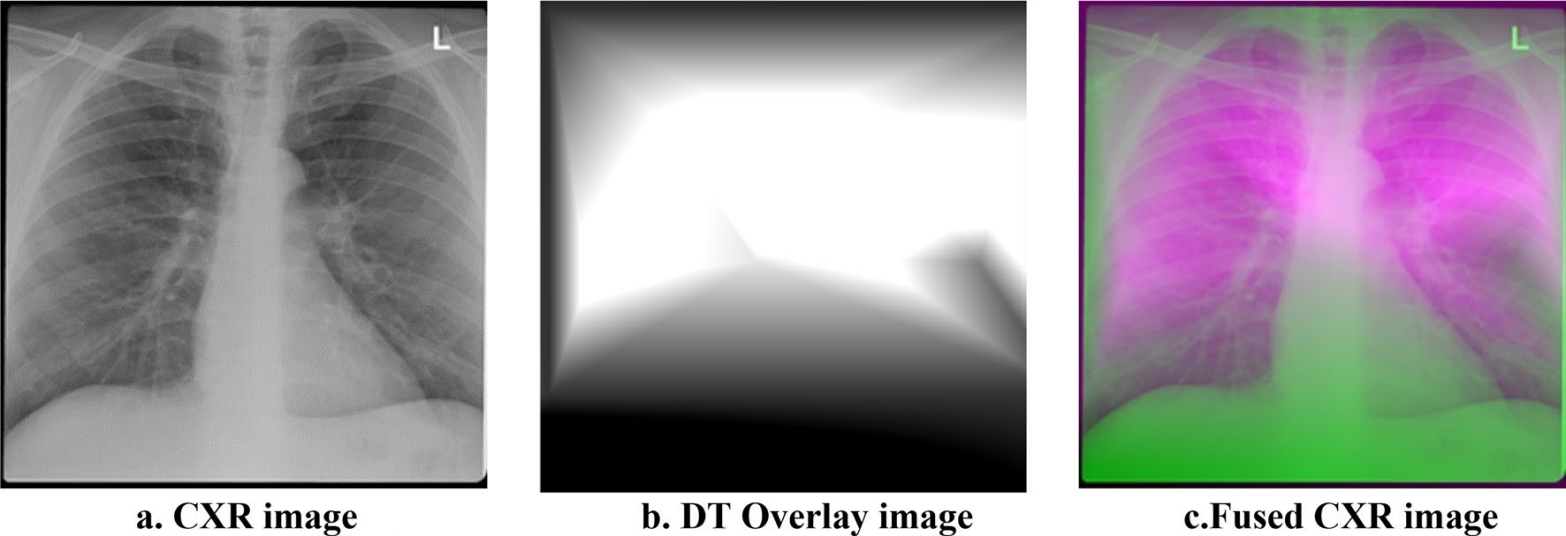
(**a**) CXR image. (**b**) DT Overlay image. (**c**) Fused CXR image.

The overlay image created utilizing the DT interpolated point from the reference points in Fig. 3 is shown in Fig. 5. Figure 6 shows the result of deep fusion. BLT interpolation’s primary goal is to reduce intra-class variation in CXR images from various data sources.

### Design of deep transfer learning neural network

The section explains the deep transfer learning model used to identify TB from an X-ray image of the chest. It is divided into two stages: choosing a Pre-Trained Model (PTM) for the provided dataset and updating the learnable layers in the chosen PTM. This section describes the three steps in designing a deep transfer learning neural network.

#### Phase 1: identification of pre-trained model for the tbXpert

The most effective PTM is chosen for tbXpert transfer learning. Two existing concepts are combined to choose the best PTM, such as revised Inception architecture and residual connections. To train deep neural networks, residual connections must be present. Employing residual connections to swap out the Inception network's filter concatenation stage is an excellent idea. As a result, residual architecture can be used while maintaining the computational effectiveness of the Inception network.

The PTM is chosen based on combining two intellectual structures: Residual connections and updated Inception architecture. Deep neural networks require residual connections to be trained. The residual connections are used to replace the filter concatenation stage of the Inception network, and it is possible to achieve the benefits of residual architecture while keeping the computational efficiency of the Inception design. To compensate for the dimensionality reduction imposed by the Inception block, the filter layer that follows each one raises the dimensionality of the filter bank. The performance of recognition has been significantly improved in the hybrid Inception-ResNet-v2 version of Inception. This PTM is used to increase accuracy for the tbXpert network.

#### Phase 2: transfer learning for tbXpert

Transferring learning (TL) uses a pre-trained model (PTM) to transfer knowledge to a similarly complex task that requires fewer data points. With 1000 image categories, the ImageNet dataset is used to train the network.

A triplet is used to represent the source domain knowledge S.

12 $$S=\left\{{I}_{s},{G}_{s},{O}_{s}\right\},$$

where ${I}_{s}$ stands for the Image Net dataset, ${O}_{s}$ for the PTM's objective prediction function, and ${G}_{s}$ for the dataset’s ground truth or label.

The target domain knowledge is signified as a triplet,

13 $${T}_{PA}=\left\{{I}_{PA},{ G}_{PA},{ O}_{PA}\right\}.$$

${G}_{PA}$ stands for the dataset's ground truths, ${O}_{PA}$ for the classifiers, and ${I}_{PA}$ for the PA view on the binary class source X-ray images.

The classifier for TL is described as,

14 $${O}_{PA}= \left\{\begin{array}{c}{O}_{PA}\left({I}_{PA},{G}_{PA }|S\right)= {O}_{PA}\left({I}_{PA},{G}_{PA }|{I}_{s},{G}_{s},{O}_{s}\right) ,\,\,with \,\,TL \\ {O}_{PA}\left({I}_{PA},{G}_{PA }\right) , \,\,without \,\,TL\end{array}\right.$$

An appropriate TL classifier reduces the prediction error,

15 $$\left[{O}_{PA}\left({I}_{PA},{G}_{PA }\right)\left(I\right), G\right]<err\left[{O}_{PA}\left({I}_{PA},{G}_{PA }|S\right)\left(I\right),G\right].$$

The foundation of transfer learning is that low-level features will be derived from the PTM's earliest levels. The target network will only be retrained for the problematic prediction task in the final layers simultaneously. The proposed methodology recognizes and removes the learnable layers while fully convolutional layers are added for accurate prediction.

**Algorithm 1:**
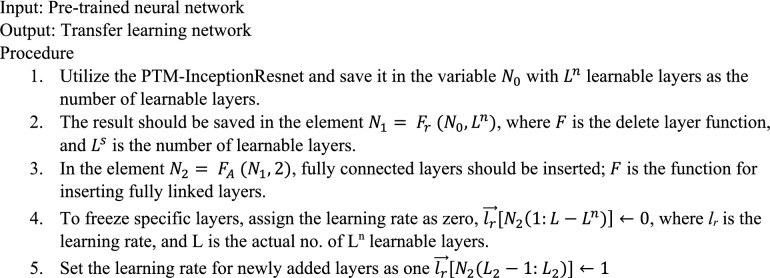
tbXpert transfer learning.

#### Phase 3: Adam classifier (adaptive movement estimation)

The appropriate optimizer to be used is to train the transfer learning neural network. Stochastic gradient descent, sometimes known as SGD, is an iterative machine learning technique that optimizes the gradient descent throughout each search after selecting a random weight vector. It is a viable solution for noisy workloads. AdaGrad and RMSPop are the most common first-order SGD algorithms. The advantages of these two methods are combined in adaptive moment estimation (Adam)^39^.

**Algorithm 2:**
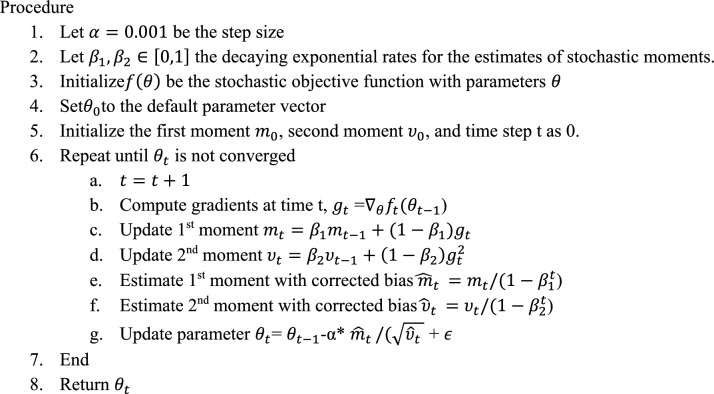
Adam optimizer algorithm for weight adjustment.

The optimized training parameter trains the proposed deep transfer learning algorithm in Algorithm 2. The model is trained to attain 100% training accuracy.

## Results

This section shows the experimental setup for implementing the proposed model tbXpert and includes the validation procedure to verify the correctness of the proposed scheme. Lastly, the recommended deep learning system named tbXpert is compared to existing cutting-edge deep learning models for diagnosing tuberculosis.

### Experimental design and analysis

**Figure 7:**
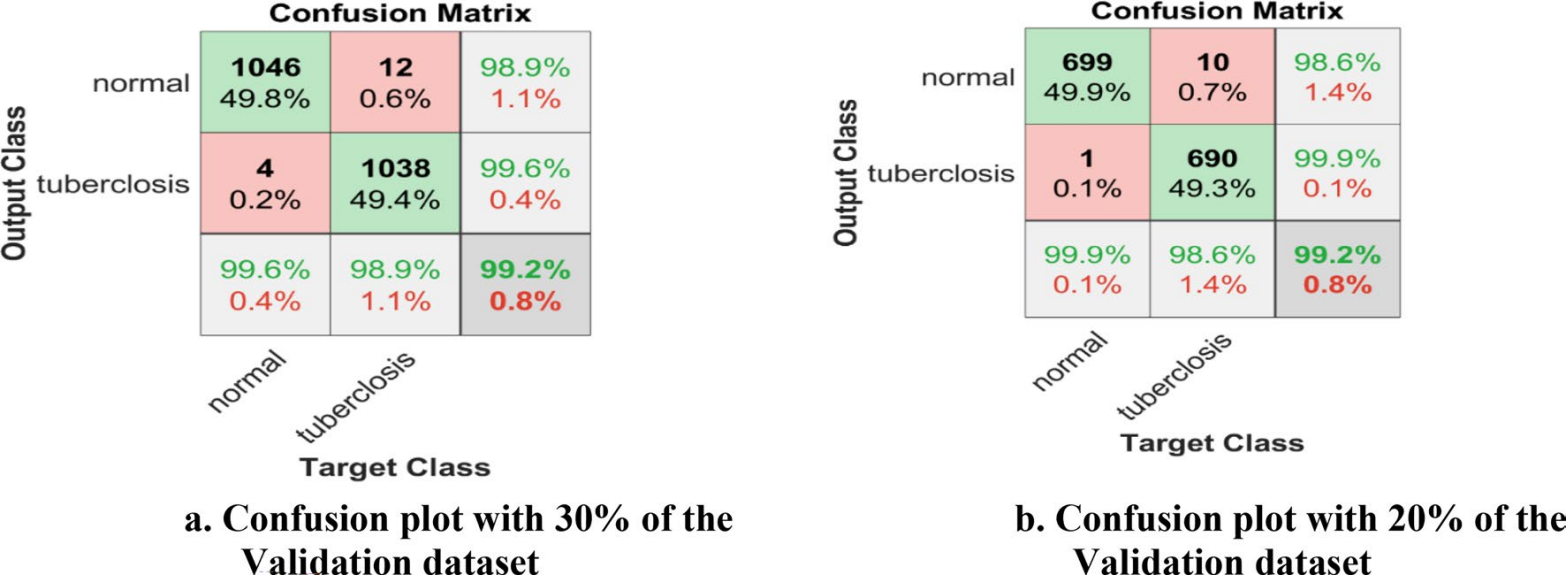
(**a**) Confusion plot with 30% of the validation dataset. (**b**) Confusion plot with 20% of the validation dataset.

The tbXpert framework was created in MATLAB 2020b using a workstation with 16 GB RAM and NVIDIA GPU. The proposed model is trained and tested with publicly available data sources such as NLM, Belarus, and NIAID, as mentioned in Sect. “Data augmentation”. The transfer learning model's hyper-parameters are adjusted for greater accuracy. Furthermore, the recommended schemes' performance is confirmed. A confusion matrix with accuracy, sensitivity, specificity, and precision is the standard metric for assessing the proposed model. Figure 7 depicts the confusion matrix with 30% of hold-out and 20% of hold-out samples.

The deep learning framework is recommended for TB prognosis. As a result, the suggested prognostic tool can be used to test for TB. The screening test can be validated by predictive power or predictive validity, a gold standard. According to the gold standard, the pediatric patient's status is positive or negative. The positive predictive value (PPV), sensitivity, negative predictive value (NPV), and specificity of screening test performance measures are estimated based on Fig. 7a. The proposed method achieves a sensitivity of 98.9%, specificity of 99.6%, precision/PPV of 99.6%, and NPV of 98.9%.

**Figure 8:**
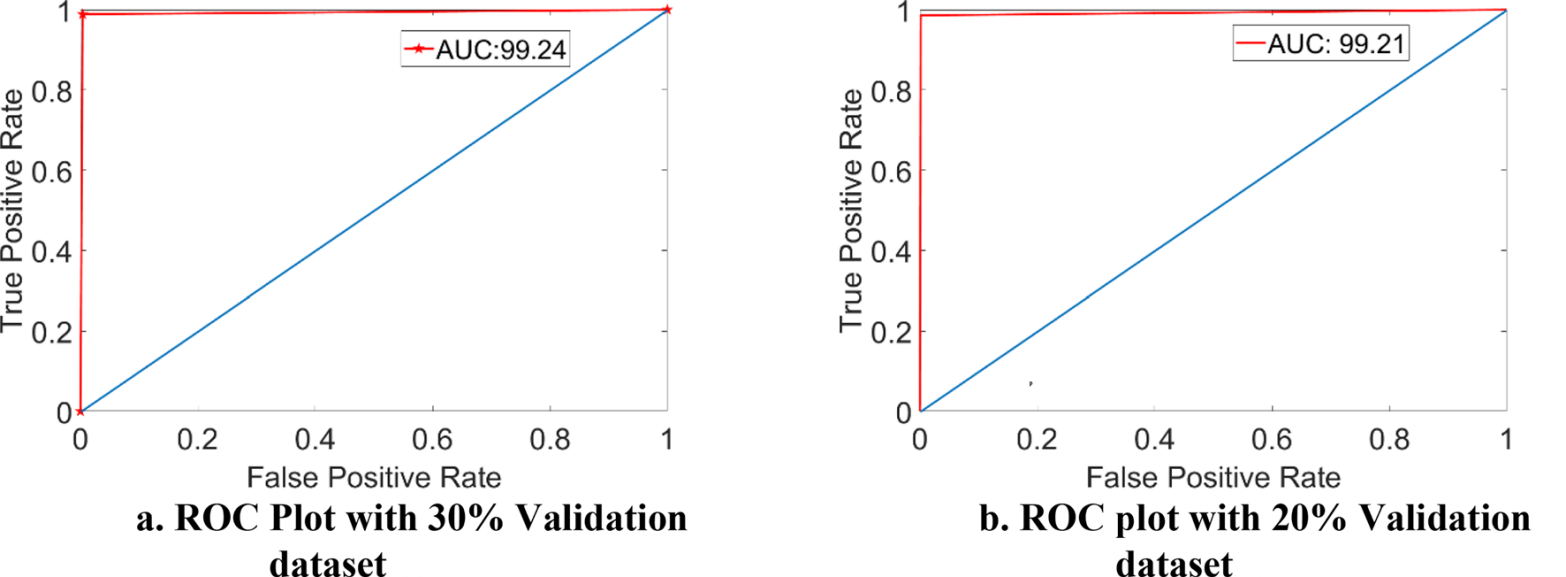
(**a**) ROC Plot with 30% validation dataset. (**b**) ROC plot with 20% validation dataset.

The Receiver Operating Characteristic (ROC) curve is a graphical technique for showing the deep learning model's performance. It is also employed to strengthen the suggested model's validity. Plotting True Positive Rate (TRP) versus False Positive Rate results in this curve (FPR). Area under the curve evaluates the general quality of the model. According to this infectious illness model, some infected cases must be appropriately identified to require prompt antibiotic treatment. Figure 8 displays the 99.2 AUC.

**Figure 9:**
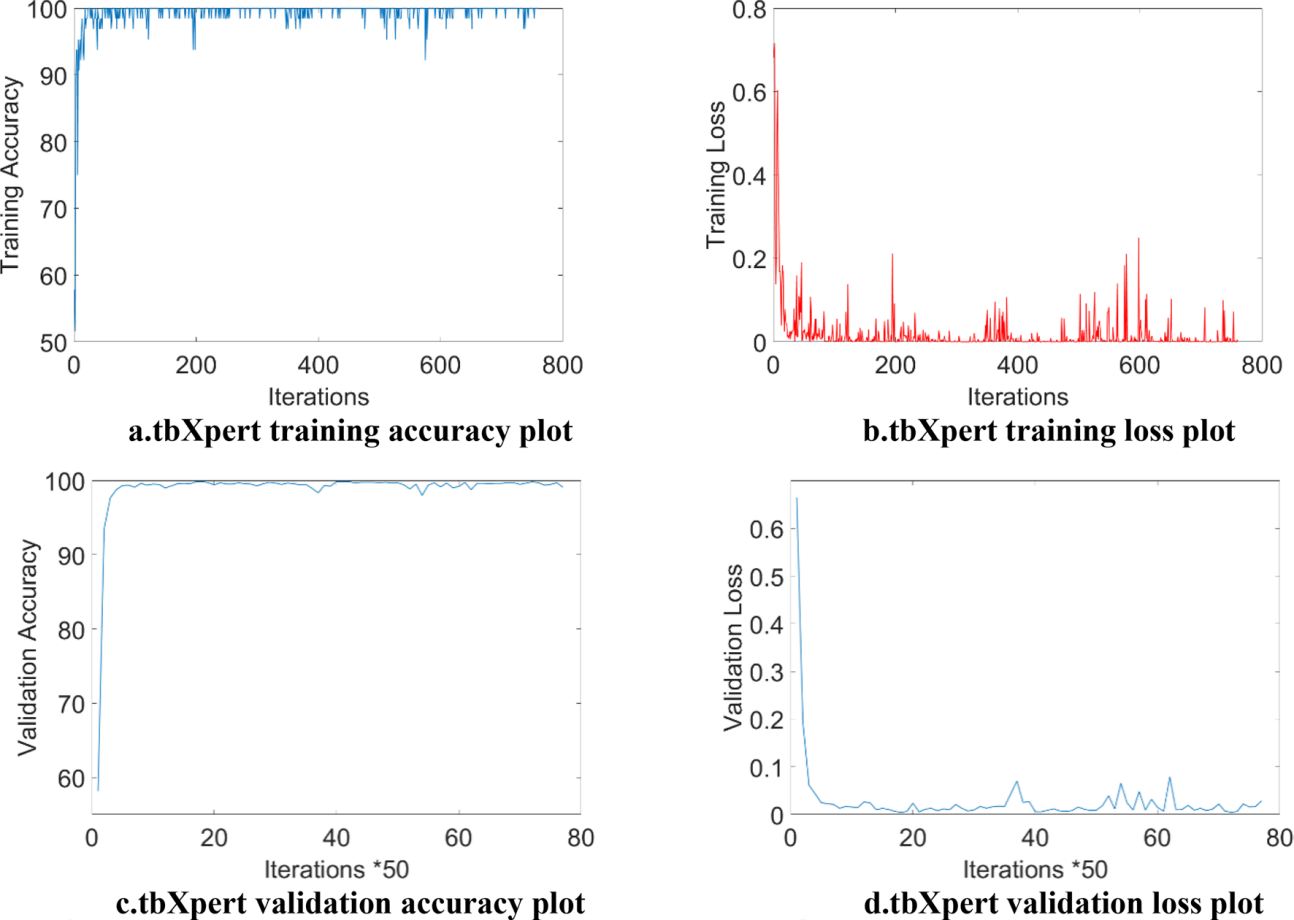
(**a**) tbXpert training accuracy plot. (**b**) tbXpert training loss plot. (**c**) tbXpert validation accuracy plot. (**d**) tbXpert validation loss plot.

The suggested model is built on the Inception network. However, it is a deep transfer learning network with residual connections. Improved training and validation results result from the residual connection's integration. Figure 9a and c illustrates that the proposed model's trianing accuracy and validation accuracy in each iterations and attained hihest validation accuracy of 99.2% after 1000 iterations, its maximum training accuracy is 100 in the first 50 iterations. Figure 9b and d display the proposed model's training loss.

### Meta-agnostic model visualization of tuberculosis

**Figure 10:**
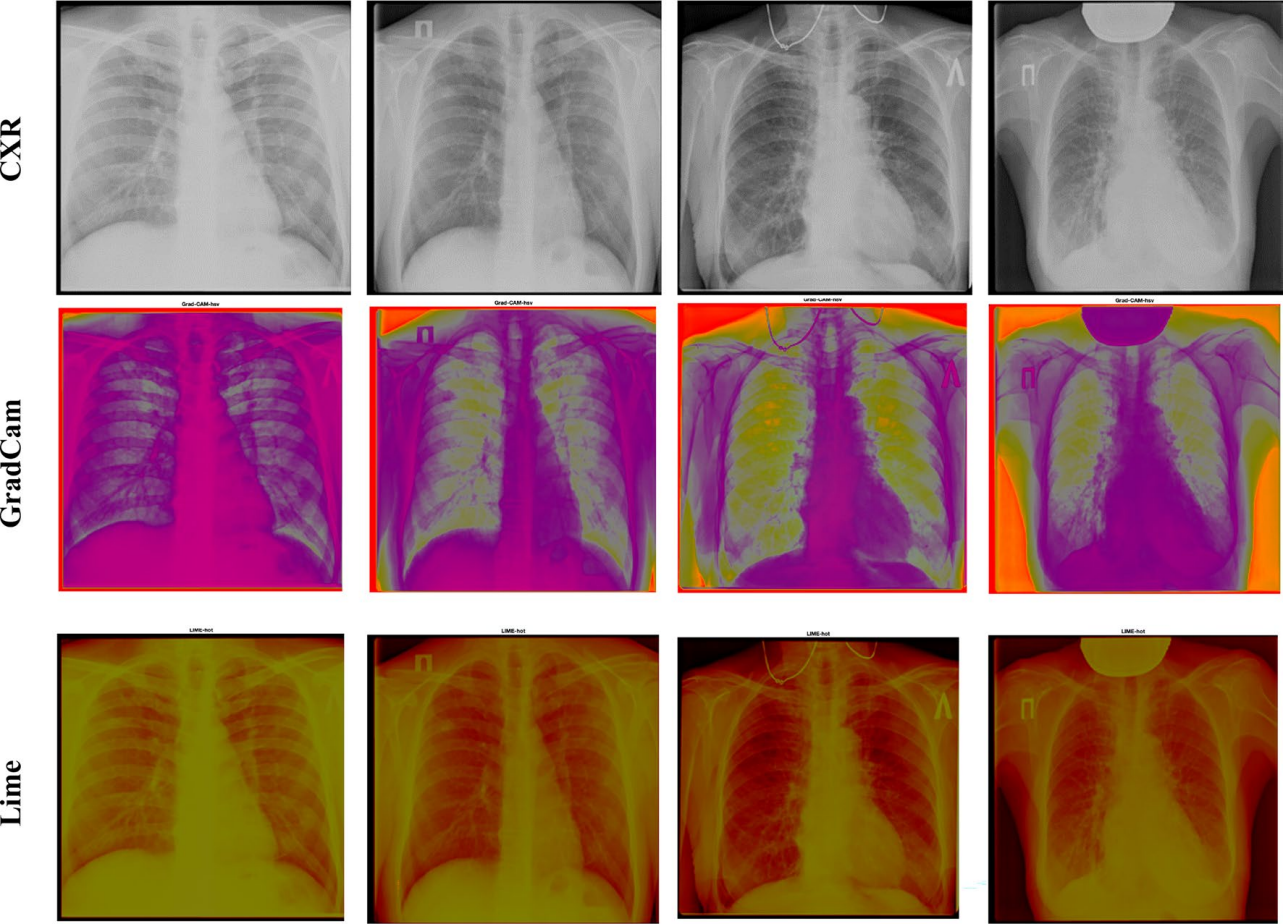
Meta agnostic model visualization of the images.

In order to provide visual explanations for the prediction results of the proposed deep neural network, Gradient-weighted Class Activation Mapping (Grad-CAM) is explored. It uses the final fully connected convolutional layer to extract a localization map with the regions for predicting the result. Grad-CAM is combined with fine-grained visualizations to construct high-resolution label-discriminative images. Figure 10 shows the Grad-CAM visualizations of the CXR images based on the constructed deep neural architecture.

The proposed deep neural network explores local Interpretable Model-Agnostic Explanations (LIME) to validate the prediction results, as shown in Fig. 10. LIME is an instance-based explainer, which generates simulated data points around an instance through random perturbation and provides explanations by fitting a weighted sparse linear model over predicted responses from the perturbed points. The explanations of LIME are locally faithful to an instance regardless of the type.

### Comparison with other models

This section compares the suggested model’s performance to existing cutting-edge methods for diagnosing tuberculosis. Researchers have recently suggested several additional techniques to identify juvenile pneumonia using chest X-ray pictures. The suggested neural network is compared to all other deep learning models for TB with the same initial data set. Table 3 displays the comparison results of the proposed model based on performance metrics such as precision, accuracy, sensitivity, and specificity.

**Table 3:** Comparison of tbXpert with other deep learning networks for TB diagnosis with the four benchmark datasets (NLM, Belarus, NIAID TB, and RSNA).

Deep convolutional neural networks	Sensitivity	Specificity	Accuracy	Precision
VGG19	95.80	95.85	95.80	95.95
ResNet18	93.85	93.91	93.85	94.08
ResNet50	93.11	93.16	93.11	93.40
ResNet101	94.55	94.59	94.55	94.74
DenseNet201	95.07	95.12	96.07	95.27
SqueezeNet	94.18	94.21	94.18	94.31
MobileNet	94.33	94.39	94.33	94.65
InceptionV3	95.73	96.51	95.72	95.92
tbXpert	98.90	99.60	99.20	99.60

**Figure 11:**
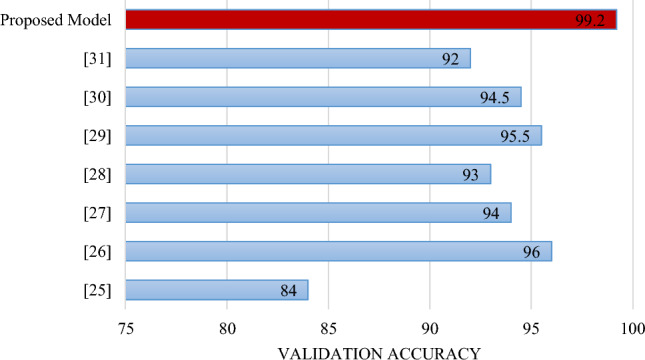
Performance comparison of the recommended model with other TB prognosis models.

Figure 11 shows that the suggested deep transfer learning method provides the highest validation accuracy of 99.2%, equating to all other TB diagnosis models. The suggested tbXpert provides the highest precision value of 99.6. It demonstrates that the proposed method outperforms all currently available deep-learning models for the prognosis of tuberculosis.

## Conclusion

A robust early TB diagnostic technique based on deep learning is suggested. In CXR images, the Linear Triangulation Interpolation reduces intraclass variation and interclass similarities. Usually, radiology images need to be more balanced in quality; therefore, the image's visual appearance must be improved to make the prognosis process more accurate. The recommended LT algorithm accurately visualizes the infected region in the image without segmentation. Inception neural network with residual connection is used to train on deep fused images. The most extensive benchmark datasets, 3500 TB CXR images and 3500 regular CXR images are used to train and evaluate the proposed tbXpert Model. Compared to other component deep learning algorithms for TB diagnosis, the designed scheme achieves validation with a sensitivity of 98.9%, specificity of 99.6%, precision of 99.6%, accuracy of 99.2%, and AUC of 99.4%, all of which are very high. To decrease the radiologist's effort and reliance on the acceptable level of competence of the specialist, the proposed tbXpert is employed as a computer-aided diagnosis approach for tuberculosis. 

## Data Availability

The datasets used and/or analysed during the current study available from the corresponding author on reasonable request.
